# BCMA-targeted CAR-T cell therapies in relapsed and/or refractory multiple myeloma: latest updates from 2023 ASCO Annual Meeting

**DOI:** 10.1186/s13045-023-01479-5

**Published:** 2023-07-28

**Authors:** James F. Wu, Binod Dhakal

**Affiliations:** 1 Department of Medicine, Medical College of Wisconsin, Milwaukee, WI USA; 2 BMT and Cellular Therapy Program, Department of Medicine, Medical College of Wisconsin, 9200 W Wisconsin Avenue, Milwaukee, WI 53226 USA

**Keywords:** CAR-T, BCMA, MM

## Abstract

Treatment of relapsed and/or refractory multiple myeloma (RRMM) utilizing the novel therapeutic target of the B-cell maturation antigen (BCMA) has demonstrated incredible results, leading to regulatory approval of BCMA-targeted chimeric antigen receptor (CAR)-T cell therapies in RRMM. With now two approved BCMA-targeted CAR-T cell therapies, investigators globally are working to build off and improve upon BCMA-targeted therapies. We discuss long-term data from the pivotal study that led to CAR-T approval, a phase 3 trial supporting their use in earlier lines, and novel manufacturing platforms to decrease vein-to-vein time. We highlight five key abstracts from the 2023 ASCO Annual Meeting that showcase these exciting updates in BCMA-directed CAR-T cell therapies in RRMM.

## To the editor

B-cell maturation antigen (BCMA)-targeted chimeric antigen receptor (CAR)-T cell therapy revolutionized treatment of relapsed and/or refractory multiple myeloma (RRMM) [1–4]. The deep and durable responses with manageable safety profiles led to US Food and Drug Administration regulatory approval of idecabtagene vicleucel (ide-cel) and ciltacabtagene autoleucel (cilta-cel) in 2021 and 2022, respectively. We summarized the important updates in BCMA CAR-T therapies for RRMM from the 2023 ASCO Annual Meeting.

### Approved BCMA-targeted CAR-T cell therapy in RRMM

Long-terms results of BCMA-targeted CAR-T trials were reported at ASCO 2023. Dr. Mi reported the ≥ 5-year follow-up data from LEGEND-2 [5], the first-in-human phase 1 study of LCAR-B38M CAR-T conducted in China, the longest follow-up for any BCMA-targeted therapy study (Table 1). Overall response rate (ORR), complete remission (CR) rate, and median progression free survival (PFS) were unchanged compared to prior reports [4]. At 65.4 months median follow-up, median overall survival (OS) was 55.8 months and 18% of patients with RRMM were alive and disease-free. Those without progressive disease were more likely to be less heavily pre-treated and have good performance status [5]. No new toxicities were reported since 48-month follow-up [4], which previously showed 9.5% grade ≥ 3 cytokine release syndrome (CRS) and one reversible grade 1 immune effector cell-associated neurotoxicity syndrome (ICANS) event (Table 2).

**Table 1:** Properties of BCMA-targeted CAR-T cell therapies in MM

Author	Agent	Clinical Trial Identifier	Phase	Target	Co-stimulatory domain/signaling domain	Mechanism	scFV	Cell source	References
Munshi et al.	ide-cel	NCT03361748	2	BCMA	4-1BB/CD3ζ	CAR-T	Chimeric mouse	Autologous	[1]
Mi et al.	cilta-cel	NCT03758417	2	Dual-binding BCMA	4-1BB/CD3ζ	CAR-T	Chimeric llama	Autologous	[3]
Mi et al.	LCAR-B38M CAR-T	NCT03090659	1	Dual-binding BCMA	4-1BB/CD3ζ	CAR-T	Chimeric llama	Autologous	[4, 5]
Lin et al.	cilta-cel	NCT03548207	1b/2	Dual-binding BCMA	4-1BB/CD3ζ	CAR-T	Chimeric llama	Autologous	[2, 6]
Rodriguez-Otero et al.	ide-cel	NCT03651128	3	BCMA	4-1BB/CD3ζ	CAR-T	Chimeric mouse	Autologous	[7]
Dhakal et al.	cilta-cel	NCT04181827	3	Dual-binding BCMA	4-1BB/CD3ζ	CAR-T	Chimeric llama	Autologous	[8, 9]
Sperling et al.	PHE885	NCT04318327	1	BCMA	4-1BB/CD3ζ	CAR-T	Human	Autologous	[10]
Du et al.	GC012F	NCT04236011; NCT04182581	1	BCMA/CD19	4-1BB/CD3ζ	CAR-T	Not specified	Autologous	[11]

*BCMA* B-cell maturation antigen, *CAR* chimeric antigen receptor, *scFV* single-chain variable fragment, *cilta-cel* ciltacabtagene autoleucel, *ide-cel* idecabtagene vicleucel

**Table 2:** Outcomes of BCMA-targeted CAR-T cell clinical trials in MM

Author	Agent	Clinical Trial Identifier	Patients (n)	Medium number of prior LOT (range)	ISS Stage 3	High-risk cytogenetics (%)	ORR (%)	≥ CR (%)	Median PFS (months)	Median OS (months)	Hazard Ratio for Disease Progression or Death	Grade ≥ 3 CRS (%)	Grade ≥ 3 Neurotoxicity (%)	References
Munshi et al.	Ide-cel	NCT03361748	128	6 (3–16)	16.0%	35.0	73.0	33.0	8.8	Immature	–	5.0	3.0	[1]
Mi et al.	Cilta-cel	NCT03758417	48	4 (3–9)	18.8%	43.8	89.6	77.1	Not reached	Not reached	–	35.4	0.0	[3]
Mi et al.	LCAR-B38M CAR-T	NCT03090659	74	3 (1–9)	28.4%	35.7	87.8	73.0	18	55.8	–	9.5	0.0	[4, 5]
Lin et al.	Cilta-cel	NCT03548207	97	6 (4–8)	14.0%	24.0	97.9	82.5	34.9	Not reached	–	5.2	12.4	[2, 6]
Rodriguez-Otero et al.*	Ide-cel	NCT03651128	254	3 (2–4)	12.0%	42.0	71.0	39.0	13.3	Immature	0.49	5.0	3.0	[7]
Dhakal et al.*	cilta-cel	NCT04181827	208	2 (1–3)	5.8%	59.4	84.6	73.1	Not reached	Immature	0.26	1.1	2.8	[8, 9]
Sperling et al.	PHE885	NCT04318327	50	4 (2–10)	54%†	36.0	98.0	41.0	–	–	–	10.0	6.0	[10]
Du et al.	GC012F	NCT04236011; NCT04182581	29	5 (2–9)	–	90.0	93.1	82.8	38	–	–	7.0	0.0	[11]

*BCMA* B-cell maturation antigen, *CAR* chimeric antigen receptor, *cilta-cel* ciltacabtagene autoleucel, *ide-cel* idecabtagene vicleucel, *LOT* lines of treatment, *ORR* overall response rate, ≥ *CR* complete response or better, *PFS* progression free survival, *OS* overall survival, *CRS* cytokine release syndrome

*Data only for CAR-T arm

^†^ISS Stage 2/3

–, not assessed by study

Dr. Lin reported the final results of CARTITUDE-1 [6], a phase 1b/2 study of cilta-cel in heavily pretreated (median 6 lines of therapy) patients with RRMM (Table 1). Median PFS was an unprecedented 34.9 months, a benefit not seen with any other therapy in this setting. At 3-year follow-up, an estimated 62.9% of patients were alive and almost half remained in remission. No new CRS or ICANS reported (Table 2). Patients will continue to be followed for safety and survival in the 15-y CARTINUE long-term study (NCT05201781; MMY4002).

### Moving BCMA-targeted CAR-T cell therapy into earlier lines

With the groundbreaking approvals of BCMA-targeted CAR-T cell therapies for RRMM, KarMMa-3 [7] and CARTITUDE-4 [8, 9] showed promise in moving these therapies into earlier lines. The phase 3 KArMMa-3 trial of ide-cel [7] in patients with 2–4 prior lines of therapy showed a 51% reduction in risk of disease progression or death versus standard of care (SOC) with a similar toxicity profile compared to previous reports with no new safety signals detected.

Dr. Dhakal presented the phase 3, randomized, controlled trial of cilta-cel [8, 9] in patients with 1–3 prior lines of therapy who were lenalidomide refractory. Cilta-cel reduced the risk of disease progression or death versus SOC by 74%, with benefits seen across all subgroups. Cilta-cel also improved ORR, rate of CR or better, and overall minimal residual disease (MRD) negativity. The side effects with cilta-cel were manageable with appropriate supportive care, and overall lower incidence and severity of CRS, ICANS, cytopenia, and other neurotoxicities were observed compared to CARTITUDE-1 (Table 2). These results are suggestive of the cilta-cel being a new standard of care for patients with lenalidomide-refractory myeloma, after the first relapse. Use in earlier LOTs is currently under evaluation in CARTITUDE-5 (NCT04923893) and Emagine/CARTITUDE-6 (EMN28; NCT05257083).

### Shorter manufacturing for BCMA-targeted CAR-T cell therapy

Several trials are utilizing novel platforms to decrease vein-to-vein time. Dr. Sperling presented the updated phase 1 results of PHE885, a BCMA-targeted CAR-T cell therapy in RRMM (Table 1) [10]. Utilization of a novel T-Charge™ platform allows for < 2-day manufacturing, with a median of 16 days from apheresis to lymphodepletion. PHE885 achieved 100% ORR at active doses with 10% grade 3 CRS and 6% grade 3 neurotoxicity (Table 2). A phase 2 study in RRMM is currently enrolling patients (NCT05172596).

Dr. Qiang presented the phase 1 results of BCMA/CD19 dual-targeting FasT CAR-T GC012F in RRMM (Table 1) [11]. The FasTCAR platform allows for 22–26 h manufacturing. In a predominately high-risk population, ORR was 93.1% with 100% MRD negativity and a median PFS of 38 months (Table 2). GC012F phase 1b/2 trials are starting in the USA and China (NCT05850234).

In conclusion, as highlighted in Tables 1 and 2, the 2023 ASCO Annual Meeting provided many innovations surrounding BCMA-targeted CAR-T cell therapy in RRMM. Translating these impressive clinical trial results, real-world access for patients will be essential.

## Data Availability

The material supporting the conclusion of this study has been included within the article.

## Abbreviations

BCMA: B-cell maturation antigen
CAR: Chimeric antigen receptor
RRMM: Relapsed and/or refractory multiple myeloma
Ide-cel: Idecabtagene vicleucel
Cilta-cel: Ciltacabtagene autoleucel
ORR: Overall response rate
CR: Complete remission
PFS: Progression free survival
OS: Overall survival
CRS: Cytokine release syndrome
ICANS: Immune effector cell-associated neurotoxicity
SOC: Standard of care
